# Supplementary material for: Orthogonal programming of heterogeneous micro-mechano-environments and geometries in three-dimensional bio-stereolithography
Source: Nat Commun. 2018 Oct 5;9:4096. doi: 10.1038/s41467-018-06685-1 (PMC6173746; doi:10.1038/s41467-018-06685-1)
Supplement: Supplementary file 1 — Supplementary Information [file 41467_2018_6685_MOESM1_ESM.docx]

Supplementary Information

**Orthogonal Programming of Heterogeneous Micro-mechano-environments and Geometries in Three-dimensional Bio-stereolithography**

Hang Yin^1*^, Yonghui Ding^1*^, Yao Zhai^1^, Wei Tan^1†^, and Xiaobo Yin^1,2†^

*^1^Department of Mechanical Engineering, University of Colorado, Boulder, CO 80309*

*^2^Materials Science and Engineering Program, University of Colorado, Boulder, CO 80309*

^*^ These authors contributed equally to this work

^†^ Correspondence and requests for materials should be addressed to W.T. (wtan@colorado.edu) and X.Y. (xiaobo.yin@colorado.edu)

Supplementary Figures 1-3

Supplementary Methods

Supplementary References

**Supplementary Figures 1-3**


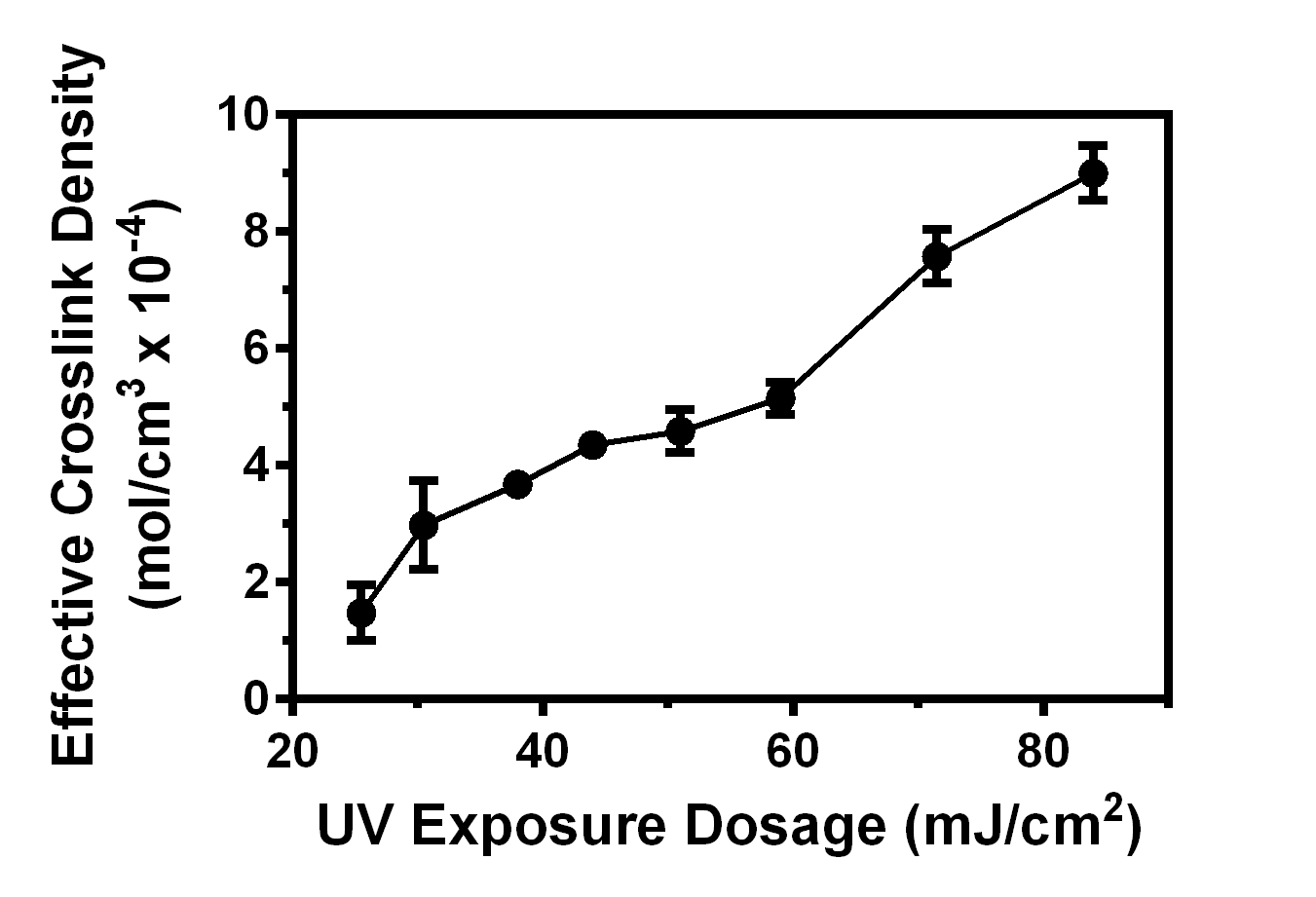


**Supplementary Figure 1. The effective crosslink density was measured by swelling experiment and Flory-Rehner calculation under different UV exposure dosage.** The results demonstrate the increasing effective crosslink density with increasing UV dosage, which is in consistent with the stiffness.


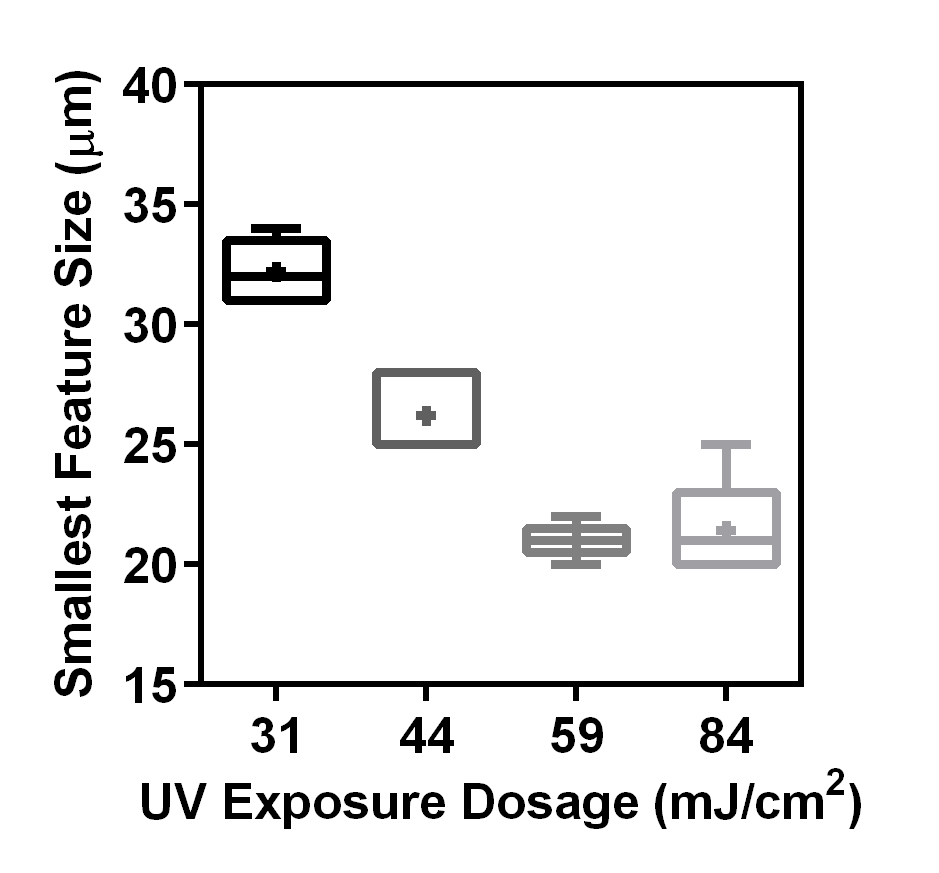


**Supplementary Figure 2. The smallest feature size that can be produced under different UV exposure dosage.** The 3D printed rods were dried, imaged by SEM, and measured by ImageJ. The smallest feature size is sensitive to the variation of stiffness (exposure dosage). A smaller feature size can be achieved with a stiff rod, which is most likely due the oxygen diffusion and inhibition. We have achieved approximately 20 μm well-defined rod structures at a dosage of 84 mJ cm^-2^.

***
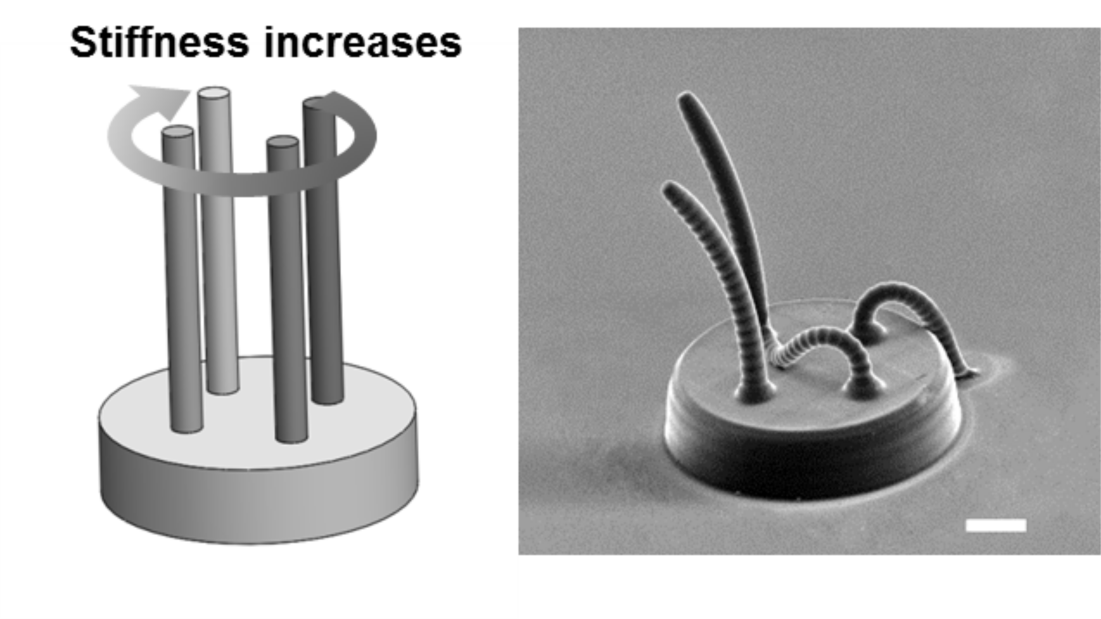
***

**Supplementary Figure 3. A demonstration of simultaneously programming multiple stiffness domains.** A 3D structure with four rods sitting on a disk which were printed with the same geometry but four spatially varied UV exposure dosages of 44, 51, 59, and 72 mJ cm^-2^. When removed from solution and dried in air, the printed four rods show different degrees of bending due to different stiffness. Left panel is schematic illustration and right panel is SEM image of printed structure after air drying. Scale bar is 200 μm.

**Supplementary Methods**

**Swelling Experiments and Flory-Rehner Calculations.** The square boxes of PEGDMA hydrogels were printed with different stiffness and then were preswollen in PBS overnight. The swollen and dried hydrogels were imaged under optical microscope to determine the swollen volume *V_s_* and dry volume *V_d_*.

We then used Flory-Rehner calculations to determine the effective crosslink density of the PEGDMA hydrogels by combining the following three equations^1,2^.

| $Q_{v}= \frac{V_{s}}{V_{d}}$ | (1) |
| --- | --- |
| $Q_{v}\cong\frac{\bar{\nu}\overline{M_{c}}}{V_{1}}(\frac{1}{2}-\chi)$ | (2) |
| $\nu_{e}= \frac{\rho_{p}}{\overline{M_{c}}}$ | (3) |

where *Q_v_* is the volumetric swelling ratio, $\bar{\nu}$ is the specific volume of the dry polymer, $\overline{M_{c}}$ is the average molecular weight between crosslinks, *V_1_* is the molar volume of the solvent (18 mol cm^3^ for water), *𝟀* is the Flory polymer-solvent interaction parameter (0.43)^3^, $\rho_{p}$ is the density of dry polymer, and $\nu_{e}$ is the effective crosslink density.

**Supplementary References**

1 Flory, P. J. *Principles of polymer chemistry*. (Cornell University Press, 1953).

2 Huglin, M. B., Rehab, M. M. & Zakaria, M. B. Thermodynamic interactions in copolymeric hydrogels. *Macromolecules* **19**, 2986-2991 (1986).

3 Merrill, E. W., Dennison, K. A. & Sung, C. Partitioning and diffusion of solutes in hydrogels of poly (ethylene oxide). *Biomaterials* **14**, 1117-1126 (1993).
